# Bilateral giant retinal tears in Osteogenesis Imperfecta

**DOI:** 10.1186/s12881-018-0521-0

**Published:** 2018-01-12

**Authors:** Paolo Scollo, Martin Paul Snead, Allan James Richards, Rebecca Pollitt, Catherine DeVile

**Affiliations:** 1 Vitreoretinal Service, Addenbrooke’s Hospital, Cambridge University NHS Foundation Trust, Hills Road, Cambridge, CB2 0QQ UK; 2 Sheffield Children’s NHS Foundation Trust, Western Bank, S10 2TH, Sheffield, UK; 3 Great Ormond Street Hospital, WC1N 3JH, London, UK

**Keywords:** Osteogenesis Imperfecta, P3H1/LEPRE1, Genetic analysis, Retinal detachment, Vitreoretinal

## Abstract

**Background:**

Osteogenesis imperfecta (OI) is a rare primarily autosomal dominant condition in which the connective tissues of bones, ligaments and sclerae do not form properly. Typically, mutations in *COL1A1* and *COL1A2* genes lead to the defective formation or quantity of type I collagen, the principle matrix in these tissues. Molecular genetic studies have now elucidated multiple genetic subtypes of the disorder but little literature exists on the risk of retinal tears and detachments in OI.

**Case presentation:**

We report the first case of a child with a rare recessive type of OI, subtype VIII, resulting from a *P3H1* (also known as LEPRE1) gene mutation presenting with bilateral giant retinal tears and the surgical challenges encountered in performing retinal detachment repair due to scleral thinning. The *P3H1* gene encodes for prolyl 3-hydroxylase 1 which is involved in the post-translational modification of not only collagen type I but also types II and V which when mutated may result in pathological posterior vitreous detachment (PVD) and giant retinal tear detachments.

**Conclusions:**

Genetic analyses are increasingly important in such cases and may guide patient monitoring and potential prophylactic treatment, known to significantly reduce the probability of giant retinal tear detachments in other high-risk collagenopathies such as Stickler Syndrome Type I.

## Background

Osteogenesis imperfecta (OI) is a rare inherited condition whereby the connective tissues of bones, ligaments and sclerae do not form properly leading to structural malformations due to the defective formation or quantity of type I collagen. Phenotypically, OI patients exhibit a range of morbidity from a mild predisposition to bone fractures to extreme bone fragility, which can be fatal in the perinatal period. The prominent ophthalmic feature of OI is ‘blue sclera’ representing scleral thinning. [1, 2].

Epidemiological and genetic studies have elucidated multiple OI subtypes demonstrating the long suspected genetic heterogeneity of OI [3]. Currently, OI diagnosis is carried out by evaluating the clinical presentation, family history, radiographic findings and laboratory investigations (molecular genetic testing or biochemical analysis of type I collagen). There are now over 15 known genetically and largely clinically distinct OI subtypes. The genes involved code for type I collagen, or factors involved in its processing, secretion and post-translational modification. Other genes causing OI affect the development or function of bone forming cells [4–6].

Previous studies describing retinal detachment (RD) in OI have not focused on the OI subtype nor underlying genetics, concentrating instead on the surgical challenges of RD repair principally due to thin sclerae, including the increased risk of scleral rupture, choroidal haemorrhage, vitreous haemorrhage and further iatrogenic RD. External approaches to RD repair in OI have been achieved via application of a sutureless scleral buckle with tissue adhesive, although this technique poses a possible risk of band dislodgement and migration [7], and application of an encircling band via sutures applied directly to muscle insertions, though this required premature buckle removal due to a recurrent conjunctival gape over the buckle site [8]. In another report, RD repairs of 4 eyes in 3 patients included demarcation laser photocoagulation alone in a macula-sparing RD, vitrectomy and gas requiring increased infusion pressure to maintain globe stability and in another case equatorial scleral buckling which was abandoned due to scleral perforation requiring conversion to vitrectomy [9]. Elsewhere, internal tamponade for RD repair in OI has been achieved via pneumatic retinopexy with successful suture closure of sclerostomies and more recently via 25-gauge sutureless vitrectomy [10–12].

## Case presentation

A 9 year-old boy with severe OI and previous history of multiple fractures, small stature and associated skull deformities presented with loss of vision in his right eye of unknown duration. The patient exhibited a low degree of myopia (RE −1.50/−3.00 × 180; LE −1.00/−3.50 × 173) and the anterior sclera was abnormally thin. Posterior segment examination revealed a right macula-off retinal detachment associated with a giant retinal tear (GRT) and C3 proliferative vitreoretinopathy (PVR).

Genetic analysis was performed by an NHS England molecular genetics service, when the child was 2 years old. At that time (2008) no potentially causative DNA change was found in COL1A1 or COL1A2, and so genetic analysis targeted regions of the CRTAP and P3H1 (previously named LEPRE1) genes, which had only recently been shown to cause recessive osteogenesis imperfecta [13]. This revealed a homozygous change c.1914 + 1G > A (NM_001243246.1) splice site mutation in intron 13 of P3H1 gene with parents being first cousins and heterozygous for same mutation. The same change has also been reported by Pepin et al. [14] in a case of osteogenesis imperfecta with compound heterozygous mutations of P3H1. Although they were unable to determine the effect of this particular c.1914 + 1G > A mutation on the mRNA, in the majority of the other cases of P3H1 mutations that they studied, mRNA instability was the outcome [14].

**Fig. 1:**
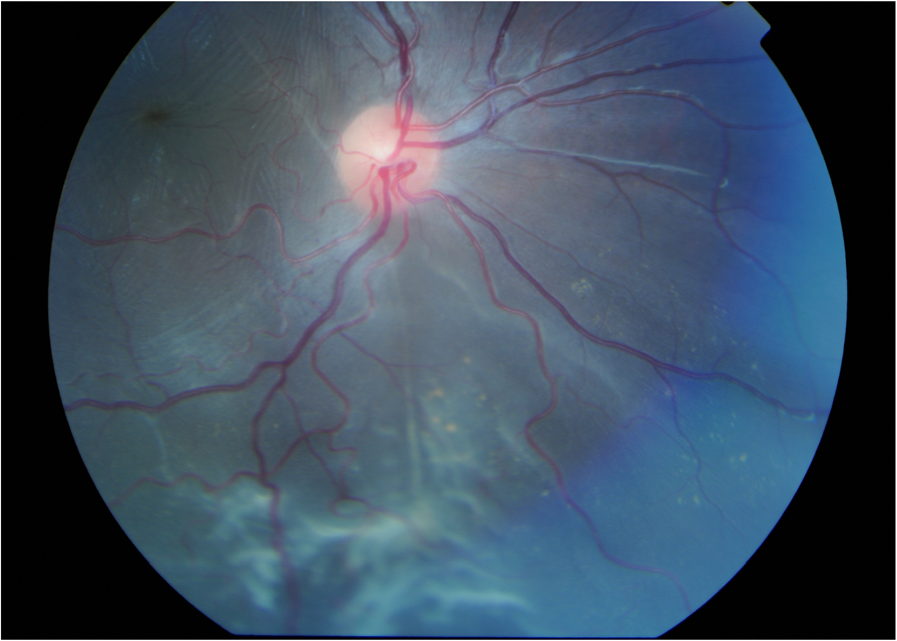
Right retinal detachment with inferior proliferative vitreoretinopathy

**Fig. 2:**
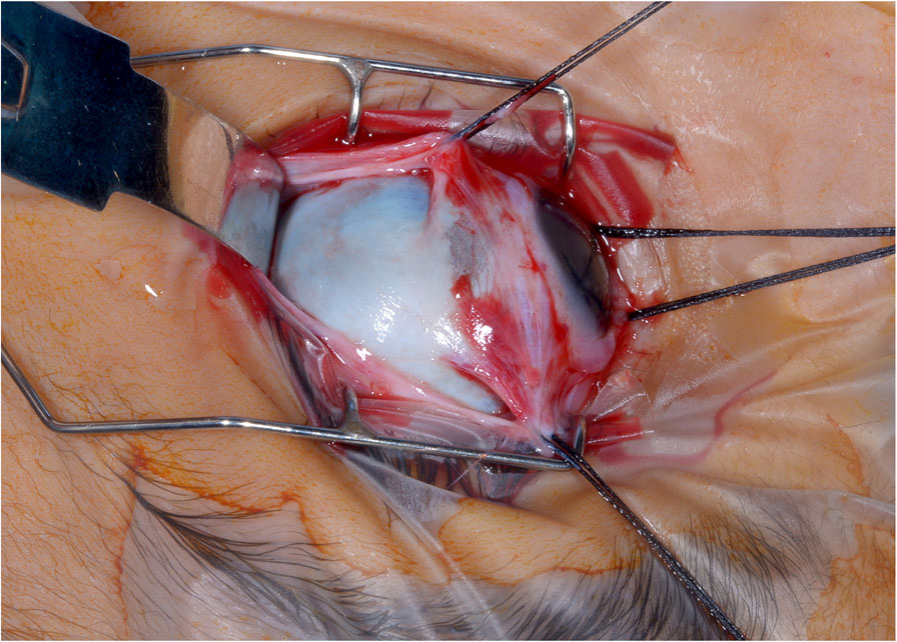
Exposed sclera following 360 degree peritomy demonstrating blue/grey hue due to scleral thinning

Examination under anaesthetic (EUA) showed a right GRT from 6 to 10 o’clock associated with a macula-off detachment and inferior epiretinal and subretinal fibrosis (Fig. 1). EUA of the fellow eye revealed a further 180-degree GRT from 1 to 7 o’clock associated with a macula-sparing retinal detachment. A right 360-degree peritomy for placement of bridle sutures revealed a sclera that was more grey than blue in hue but very thin (see Fig. 2). Right RD repair was performed via single-port pars plana vitrectomy (PPV), tamponade with silicone oil and cryoretinopexy extended to 360 degrees. The single sclerostomy and conjunctiva were successfully closed with polyglactin absorbable sutures.

Surgery for the left eye consisted of PPV and fluid/perfluorocarbon liquid (PFCL) exchange to unroll the posterior flap of the GRT, followed by direct PFCL-silicone oil exchange. Retinopexy was again applied to both the GRT and completed through 360 degrees.

Persistent antero-posterior PVR meant further right eye surgery was required, involving a 360-degree buckle, a silicone oil top-up and further retinopexy to seal a leak in the lower horn of the GRT. Scleral fixation sutures were not possible for the buckling procedure, which was completed with two 5/0 vicryl sutures tethering the anterior edge of the buckle to the medial and lateral recti to prevent posterior buckle migration with end to end shortening and suturing of the 360-degree explant. Retinal stability was achieved in both eyes but a chronic inferior RD in context of extensive PVR remains under silicone oil in the right eye. Visual acuity with silicone oil in situ at time of writing is RE 6/36, LE 6/12.

## Discussion and conclusions

Some suggest that decreased ocular rigidity in OI could increase tractional forces on the peripheral retina placing greater stress upon the vitreoretinal-retinal pigment epithelium relationship, possibly increasing the risk of RD [10]. Ocular rigidity is significantly reduced in OI when compared with controls but this fails to explain the causal relationship with pathological PVD leading to GRTs. Madigan et al. [10] speculate that if collagen type I is affected in OI, collagen type II could also be affected by some unknown mechanism. To our knowledge, no subsequent studies have investigated the underlying genetic mechanisms that result in OI patients having a predilection for pathological PVD leading to RD.

The P3H1 gene, abnormal in OI subtype VIII, encodes for prolyl 3-hydroxylase 1 which is involved in catalysing the post-translational modification of not only collagen type I found in the sclera but also collagen types II and V which are expressed in the mature vitreous matrix [15]. We hypothesise that the case described above raises the possibility of a common pathway shared with other vitreoretinopathies affecting collagen sub-types expressed in the vitreous, including *COL2A1* and *COL11A1* gene mutations that cause defective type II and XI vitreous collagen in Stickler Syndrome, also associated with a high risk of pathological PVD and GRT detachment [16].

Earlier studies describe type VIII OI as a phenotype typically expressing normal sclerae [15], however, the anterior sclerae in our patient was found to be abnormally thin. There is a high risk of scleral perforation with scleral buckling techniques in OI even when sutures are placed superficially and as found in this case, as well as in a previous report, a lack of scleral blueness may be misleading [9]. Although successful approaches to RD repair in OI can be achieved via sutureless micro-incision vitrectomy, an approach with potentially shorter operating times, reduced trauma, astigmatism and patient discomfort [12], the potential greater risk of wound leakage, hypotony and intraocular infection in patients with very thin sclera must be considered.

RD can be devastating to vision and in OI in particular may cause further morbidity due to a greater risk of bone fractures. In OI, genetic analysis may potentially guide ophthalmic monitoring programmes and where appropriate prophylactic treatment which has been shown to significantly reduce the probability of RD in other high-risk genetic conditions such as Stickler Syndrome Type I [16]. Further research is required to elucidate the molecular mechanisms of pathological PVD in such patients.

## Abbreviations

EUA: Examination Under Anaesthetic
GRT: Giant retinal tear
LE: Left Eye
OI: Osteogenesis imperfecta
PFCL: Perfluorocarbon liquid
PPV: Pars Plana vitrectomy
PVD: Posterior vitreous detachment
PVR: Proliferative vitreoretinopathy
RD: Retinal detachment
RE: Right eye
